# Facile ZnO-based nanomaterial and its fabrication as a supercapacitor electrode: synthesis, characterization and electrochemical studies

**DOI:** 10.1039/d1ra04341b

**Published:** 2021-07-02

**Authors:** Irum Shaheen, Khuram Shahzad Ahmad, Camila Zequine, Ram K. Gupta, Andrew G. Thomas, Mohammad Azad Malik

**Affiliations:** Department of Environmental Sciences, Fatima Jinnah Women University Rawalpindi Pakistan chemist.phd33@yahoo.com dr.k.s.ahmad@fjwu.edu.pk; Department of Chemistry, Pittsburg State University 1701 South Broadway Street Pittsburg KS 66762 USA; Department of Materials, Photon Science Institute and Sir Henry Royce Institute, Alan Turing Building The University of Manchester Oxford Road Manchester M13 9PL UK

## Abstract

In recent times, tremendous efforts have been devoted to the efficient and cost-effective advancements of electrochemically active metal oxide nanomaterials. Here, we have synthesized a facile nanomaterial of ZnO@PdO/Pd by employing extracted fuel from *E. cognata* leaves following a hydrothermal route. The phyto-fueled ZnO@PdO/Pd nanomaterial was fabricated into a supercapacitor electrode and was scrutinized by galvanostatic charge–discharge, electrochemical impedance spectroscopy and cyclic voltammetry to evaluate its energy storage potential, and transport of electrons and conductivity. Substantial specific capacitance *i.e.*, 178 F g^−1^ was obtained in the current study in aKOH electrolyte solution. A specific energy density of 3.7 W h Kg^−1^ was measured using the charge–discharge data. A high power density of 3718 W Kg^−1^ was observed for the ZnO@PdO/Pd electrode. Furthermore, the observed low internal resistance of 0.4 Ω suggested effective electron- and ion diffusion. Thus, the superb electrochemical behavior of the ZnO@PdO/Pd nanocomposite was exposed, as verified by the significant redox behavior shown by cyclic voltammetry and galvanostatic charge–discharge.

## Abstract 2

In recent times, tremendous efforts have been devoted to the efficient and cost-effective advancements of electrochemically active metal oxide nanomaterials.

## Graphical abstract

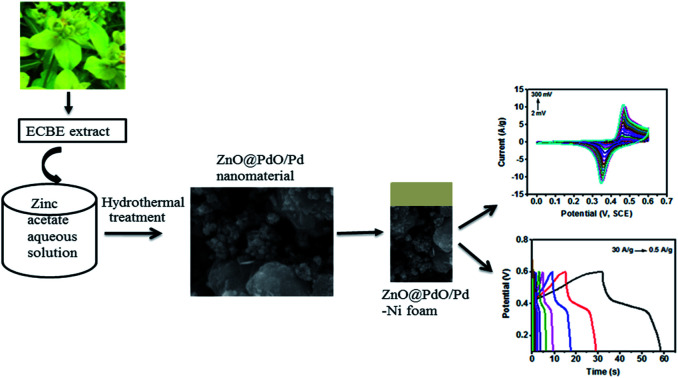

## 1. Introduction

The current era of advancement in technology has greatly increased the demand of electrochemical advanced nanomaterials, such as supercapacitors, batteries, and fuel cells.^1–6^ The performance of these devices critically depends on the flow of electrons or conductivity of the electrode material.^6,9,15–18^ Therefore, numerous electrode materials have been intensively investigated among the scientific community to enhance the performance of electrodes.^7–18^ It is believed that a straightforward approach to develop an efficient electrode is the functionalization of nanomaterials.^19,20^ Nano-sized materials contain more active sites, which thus enhance the electronic and ionic conductivity of an electrode.

Numerous studies have been carried out on diverse nanomaterials with higher electro-activity to adapt the efficiency of supercapacitors.^15–18^ The carbon-based nanomaterials are being investigated for electrical double layer capacitors as these capacitors materials have a higher pore size and surface area. Then, the next category of nanomaterials is transition metal oxide nanomaterials used in the fabrication of pseudocapacitors, which present outstanding specific capacitance and energy density.^17,18^ An electrical double layer capacitor shows charge storage by reversible ion adsorption at the surface of the electrode and electrolyte interfaces, while charge storage shown by pseudocapacitors is *via* Faradaic-redox reactions occurring at the electrode surface. Pseudocapacitors due to their fast reactions exhibit higher energy density and higher specific capacitance compared to EDLCs. In pseudocapacitors, electrons are passed on the valence band of the anode species or a redox cathode. Therefore, pseudocapacitors rest on the nature and the structure of the electrode material.^18–20^

Among various pseudocapacitor electrode materials, transition metal oxides (TMOs) with numerous valences are considered as the best electrode materials for pseudocapacitors, offering an enhanced oxidation states for the effective redox ion transfer. Among TMOs, ZnO is one of the most suitable materials for pseudocapacitor applications due to its higher electrochemical activity, lower cost and greener nature. Recently, we synthesized ZnO NPs using the organic fuel prepared from leaf extract of *Euphorbia cognata Boiss*, and evaluated its possibility for energy storage devices.^5^ Phyto-mediated ZnO NPs revealed a specific capacitance of 86 F g^−1^ at 2 mV s^−1^.^5^ Then, we have synthesized and reported the phyto-assisted ZnO–Co_3_O_4_ as an electrode material for supercapacitors, and this electrode material showed a capacitance of 165 F g^−1^.^1^ Another, ZnO-based electrode material, ZnO@NiO, was synthesized *via* the same biogenic route using the leaf extract of *Euphorbia cognata Boiss.*^3^

Thus, the reported investigations vividly showed that the leaf extract of *Euphorbia cognata Boiss* (hereafter written as ECBE) was a significant reagent for the synthesis of metal oxides nanomaterials.^1,3,5,21,22^ Motivated from the success of pervious projects of ECBE-based nanomaterials, we have employed the same ECBE-based synthesis strategy to synthesize a facile nanomaterial of ZnO@PdO/Pd without using any chemical reagent. The ZnO-based nanomaterial possessed high crystallinity, and thus possessed good supercapacitive properties.^1–5^ In this study, supercapacitive properties of the facile ZnO@PdO/Pd nanomaterial have been investigated by galvanostatic charge–discharge (GCD), electrochemical impedance spectroscopy (EIS) and cyclic voltammetry (CV) techniques in a non-toxic and lower cost aqueous electrolyte, and it showed considerable specific capacitance.

The present study is first a comprehensive exploration carried out on the preparation of a phyto fuel (PF)-doped mixed metal oxide (MMO) nanostructure, and the recognition of phyto-stabilizing agents (PSAs) in the obtained product along with the exploration of its electrochemical performance related to supercapacitors. The electrochemical properties of the as obtained novel nano-product were tested at various scan rates ranging from higher (300 mV s^−1^) to lower (2 mV s^−1^) and under numerous current densities (0.5–30 A g^−1^). The overall electrochemical results revealed that the ZnO@PdO/Pd nanomaterial was a capable material useful as an electrode for supercapacitor applications.

## 2. Material and methods

Zinc acetate [Zn(O_2_CCH_3_)_2_ (H_2_O)_2_] and palladium acetate [(Pd(CH_3_COO)_2_)], ethanol and methanol (C_2_H_5_OH) were bought from Sigma Aldrich, Germany. The electrode material fabrication was carried out using acetylene black, polyvinylidene (PVDF) as well as *N*-methyl pyrrolidinone (NMP) bought from Sigma Aldrich, Germany. Fresh leaves of *E. cognata* (*EC*) were obtained from Rawalakot area [33.8584° N, 73.7654° E] of AJK, Pakistan.

### 2.1. Preparation of the ZnO@PdO/Pd nanomaterial

**Fig. 1:**
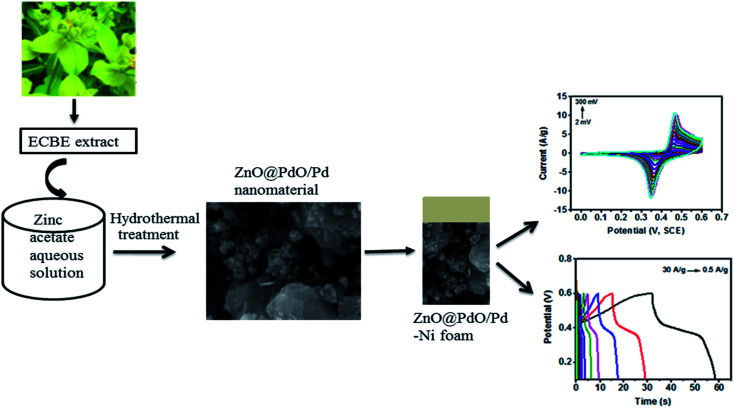
PF-supported preparation of the ZnO@PdO/Pd nanomaterial.

The ECBE was prepared in DI, and its preparation has been reported in recent studies.^1,3,5^ Strategy utilized in this study is depicted in Fig. 1. The ZnO@PdO/Pd nanostructure was fabricated using ECBE. First, 40 mM *o*f Zn(O_2_CCH_3_)_2_ (H_2_O)_2_ was prepared by dissolving 878 mg of Zn(O_2_CCH_3_)_2_ (H_2_O)_2_ into 100 mL of DI solvent, *i.e.*, 3.99 moles of Zn(O_2_CCH_3_)_2_ (H_2_O)_2_ were dissolved in 100 mL of DI water inside a 200 mL conical flask. 10 mL of ECBE was then taken from the stock extract, and dropped in a conical flask at a magnetic hot plate. The reaction was stirred for almost 2 h at about 70 °C. Incubation was carried out for this resultant mixture for almost 24 h. Then, a desiccation treatment was conducted first on a hot air oven at 95 °C and then by annealing on an air-furnace at about 450 °C for almost 4 h for attaining fully functionalized ZnO NPs, which were then sonicated in DI water using an ultrasonicator.

Hereafter, 40 mM [(Pd(CH_3_COO)_2_)] solution was prepared by taking 898 mg (4 moles) of [(Pd(CH_3_COO)_2_)] into 100 mL of DI water. This sonicated suspension of ZnO NPs was added into the prepared 40 mM solution of [(Pd(CH_3_COO)_2_)] in a ratio of 8:2 *via* a stirring (550 rpm) treatment for about 15 min along with temperature upsurged up to 70 °C. Moreover, 2.5 mL of ECBE was also mixed in this mixture along with stirring for another 1 h. The mixture was then evaporated within an oven for all night, and then dried in a furnace at 450 °C for 4–5 h. The resultant material was ground, saved in Eppendorfs, and labelled as ZnO@PdO/Pd.

## 3. Characterization

ZnO@PdO/Pd was analysed *via* UV-Vis spectrophotometry (UV-Vis, spectrophotometer 1602, Biomedical services, Spain) to study the bandgap. OGFs were monitored *via* FT-IR spectroscopy (FTIR, 8400, Shimadzu, Japan). Presence of organic species was confirmed by GCMS QP5050. Phases were identified by XRD (XRD5 PANalytical X'Pert Pro, Manchester, U.K.). The shape and type of elements present were determined by SEM (Quanta 250-FEG, Thermo Fisher Scientific, U.S.A.) along with EDX (Bruker, U.S.A). Similarly, investigations on the surface chemistry were accomplished *via* a Kratos-Axis Ultra spectrometer (XPS).

### 3.1. Electrochemical measurements

In order to fabricate the resultant product, within *N*-methyl pyrrolidinone, a slurry encompassing ZnO@PdO/Pd, AB, as well as PVDF in 8 : 1 : 1 was prepared, which was then dispersed over porous Ni foam at 60 °C for 10 h under vacuum. The electrode material was examined electrochemically *via* CV, GCD, and EIS. To carry out these studies, an electrochemical work station was produced having a three-electrode system along with a working electrode comprising a ternary nanocomposite-adapted Ni foam, a platinum wire counter electrode and saturated calomel as the reference electrode. Electrochemical analyses were carried out *via* KOH (3 M) by *CV* at varying scanning rates ranging 2–300 mV s^−1^. Galvanostatic charge discharge (GCD) was altered by fluctuating the current densities (CD) *i.e.*, 0.5–30 A g^−1^. *EIS* was directed for sample utilizing frequency of 50 mHz to 10 kHz.

## 4. Results and discussion

### 4.1. Functionalized ZnO@PdO/Pd nanomaterial

In the literature, numerous plant-mediated nanomaterials have been synthesized.^23–28^ In the current study, a facile nanomaterial has been synthesized for investigating its electrochemical behaviour. Before employing ZnO@PdO/Pd for fabrication of an electrode, it was analysed for its compositional and morphological properties. Initially, PF-assisted ZnO@PdO/Pd was examined by FTIR spectroscopy and GC-MS for obtaining the presence of PSAs.

**Fig. 2:**
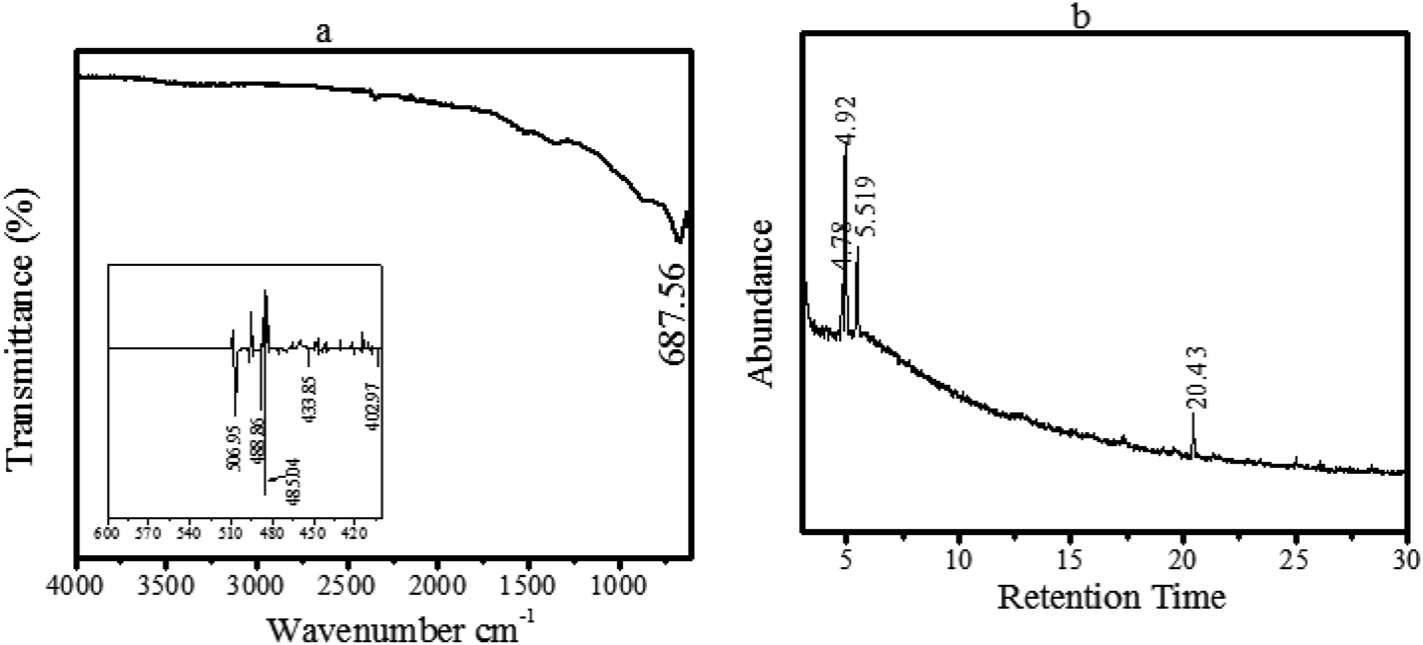
(a) FTIR spectra of the as-prepared ZnO@PdO/Pd, (b) GC-MS chromatogram of the ZnO@PdO/Pd.

Fig. 2a shows the FTIR analysis of our observed material (ZnO@PdO/Pd), displaying clear vibrational peaks at 687.56 cm^−1^, given by the N–H wag along with the C–H oop, matching to aromatics as well as 1° and 2° amines. These vibration peaks were associated with the PF-doped ZnO@PdO/Pd material. Inset shown in Fig. 2a reflects M–C, and M–O (M = Zn and Pd) bonds within frequency 600–400 cm^−1^.^39,54^ Thus, aromatics and 1° and 2° amines behaved as SAs in the as-prepared product.

GC-MS of our tested sample is presented in Fig. 2b, showing peaks at 4.78, 4.92, 5.519, as well as at 20.43, representing benzeneethanamine (C_8_H_11_N), benzenemethanol (C_7_H_8_O), benzeneethanamine along with octodrine (C_8_H_19_N), connected to PdO/Pd-doped ZnO, respectively.

**Fig. 3:**
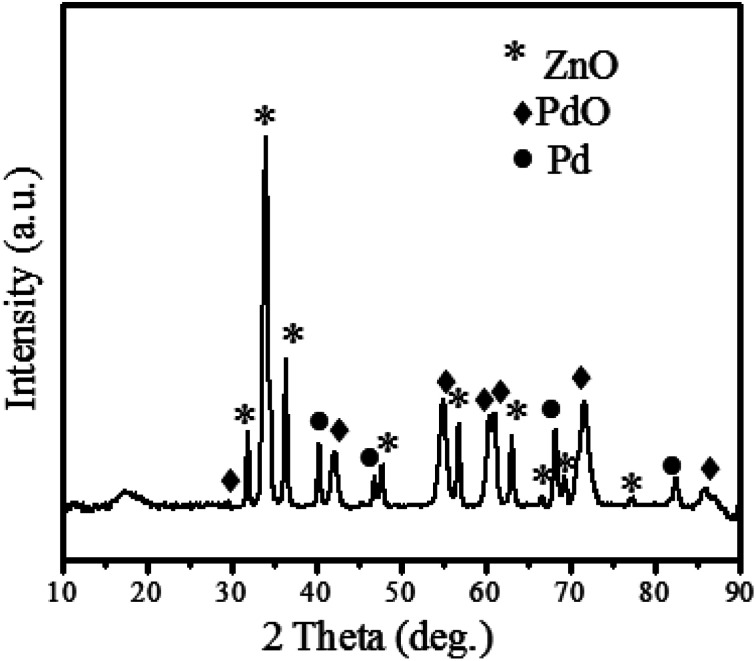
X-ray diffractogram of the ZnO@PdO/Pd nanomaterial.

Fig. 3 shows the XRD patterns of hexagonal ZnO according to (ICSD 00-036-1451). ZnO showed crystalline peaks at 2 theta (*θ*) = 31.97, 34.65, 36.465, 47.751, 56.79, 63.06, 66.63, 69.24, and 77.128, relating to (100), (002), (101), (102), (110), (103), (200), (201) and (202) miller index planes. According to ICSD 00-036-1451, the crystal system is hexagonal for ZnO, the space group is *P*6_3_*mc* and space group number is 186. The unit cell parameters are as follows;✓*a* (Å): 3.2498✓*b* (Å): 3.2498✓*c* (Å): 5.2066✓Alpha (°): 90✓Beta (°): 90✓Gamma (°): 120

Moreover, XRD revealed the PdO (♦) at 2 theta (*θ*) = 34.082, 42.184, 54.97, 60.45, 71.61, and 85.81 corresponding to *hkl* planes of (101), (110), (112), (103), (211), and (114) (ICSD 00-041-1107). According to the ICSD 00-041-1107 crystal system, PdO is tetragonal, its space group is *P*4_2_/mmc, space group number is 131, and the unit cell parameters are *a* (Å): 3.0456, *b* (Å): 3.0456, *c* (Å): 3.3387, alpha (°): 90, beta (°): 90, gamma (°): 90. However, the formation of cubic Pd (●) was confirmed by peaks at 2 theta (*θ*) = 40.3601 (111), 46.89 (200), 68.22 (220), 82.35 (311) and 86.06 (222) with ICSD 00-005-0681. However, the formation of cubic Pd (●) was confirmed by peaks at 2 theta (*θ*) = 40.3601 (111), 46.89 (200), 68.22 (220), 82.35 (311) and 86.06 (222) with ICSD 00-005-0681. ICSD 00-005-0681 states that the crystal system of Pd is cubic, space group is *Fm*3*m*, space group number is 225, while unit cell parameters are *a* (Å): 3.8898, *b* (Å): 3.8898, *c* (Å): 3.8898, Alpha, Beta and Gamma is same as for PdO. Moreover, the crystallite size shown by ZnO@PdO/Pd was 27–29 nm as calculated by the Debye Scherrer's.^71^

**Fig. 4:**
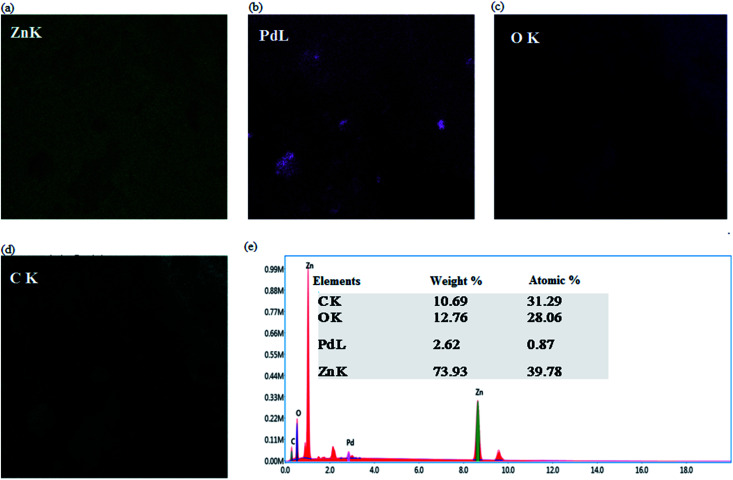
(a–d) Elemental profiling of the as-synthesized nanomaterial, (e) EDX spectrum of the as-synthesized material.

EDX analysis of the tested sample is given in Fig. 4, indicating that Zn was in excess, whereas Pd was in a minor quantity. Results further verified the carbon presence, which is because of PSAs.

**Fig. 5:**
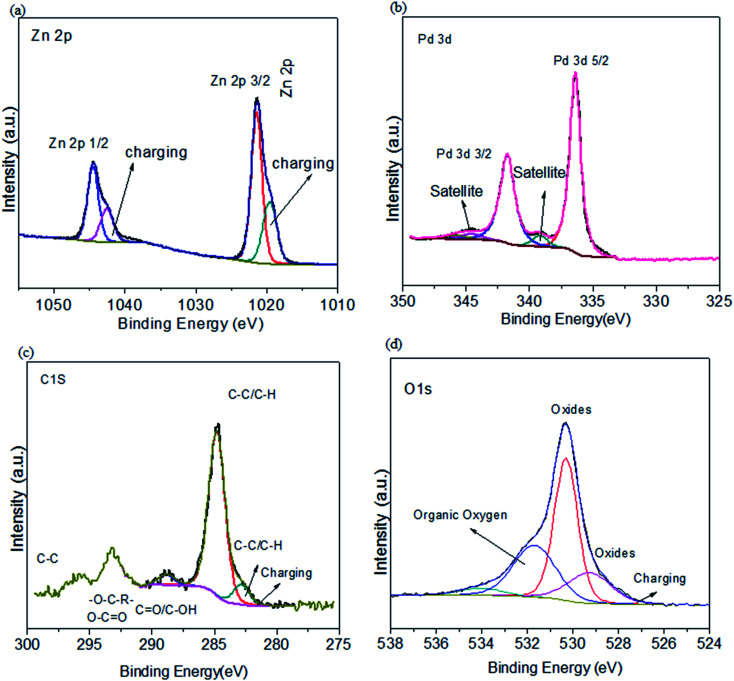
XPS of ZnO@PdO/Pd (a) Zn 2p, (b) Pd 3d, (c) C 1s, (d) O 1s.

The Zn 2p spectrum (Fig. 5a) shows two orbit split peaks by Zn-2p_3/2_ along with Zn-2p_1/2_,^39^ with binding energies (BE) relatable to the ZnO spectra.^42^ Pd 3d spectra indicate that Pd 3d_5/2_ and Pd 3d 3/2 also have Pd^2+^ on 342.2 BE (Fig. 5b). The O1s region (Fig. 5d) indicates C=O, C–O, OH and H–C–O.^29,42^ The C 1s spectrum shows OFGs, *i.e.*, C–O, COO, O–C=O, C=O, C–C, C–H and C=C.^29,42,64^ The energy region in C 1s is higher than 290 eV because of sp^3^ hybridized (C–C).^64^ Thus it is fruitfully expressed by XPS that the functionalization of ZnO@PdO@Pd by OFGs is achieved in a good way.

**Fig. 6:**
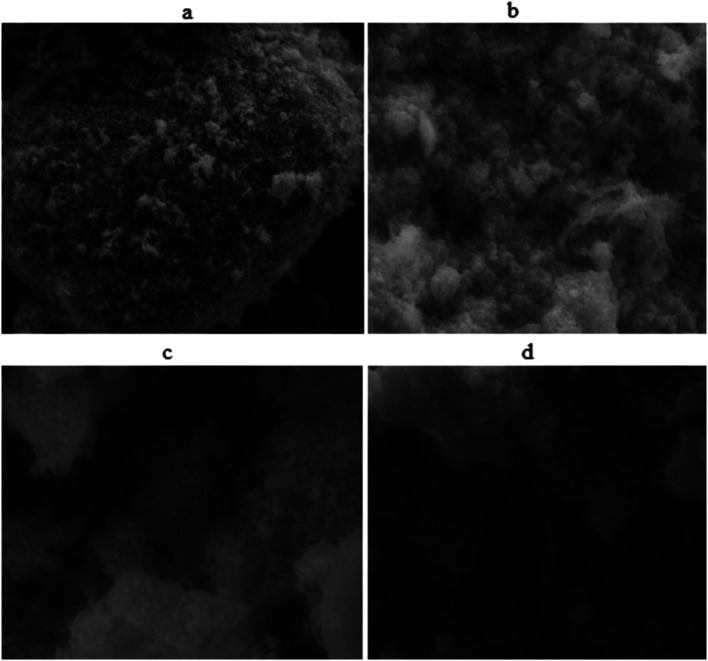
Morphological images ZnO@PdO/Pd at different magnifications (*a* = 5 μm, *b* = 3 μm, *c* = 1 μm and 500 nm).

FE-SEM pictures of ZnO@PdO/Pd displayed in Fig. 6 gave distinct nano-structures in spherical forms arranged in an even and less agglomerated fashion, thus verifying that the role of PF for the preparation of required material is done in an excellent way.^1,3,5,23–25^

### 4.2. Bandgap energy

**Fig. 7:**
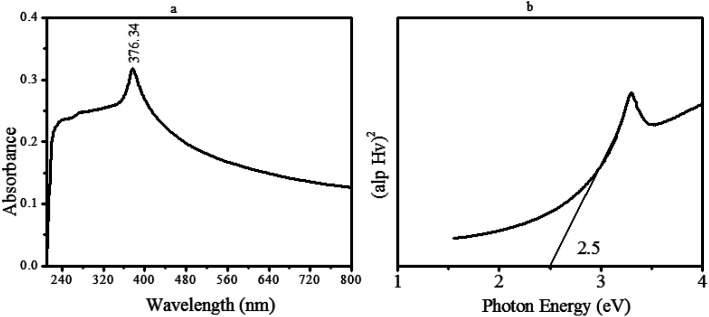
(a) UV Absorption analysis displayed by ZnO@PdO/Pd, (b) optical bandgap energy (BGE) of ZnO@PdO/Pd through Tauc's plot.

Apart from compositional and morphological analysis, the facile fabricated material was further investigated for its optical direct bandgap energy (eV). For this, a very diluted and transparent aqueous suspension of the as-synthesized nanomaterial was scanned by UV-Vis at 200–800 nm. The subsequent spectrum is displayed in Fig. 7a. Based on the absorbance data and using Tauc's equation, the optical bandgap energy was determined (Fig. 7b).

Fig. 7a depicts an absorption peak at 376.34 nm related to OFGs and MOs correspondingly. Fig. 7b shows the BGE of ZnO@PdO/Pd through Tauc's plot. Consequential BGE for OFG-binded ZnO@PdO/Pd was very low, *i.e.*, 2.25 eV, which made the transference of electrons quite easy with greater CD making it promising for capacitance.^29–31,43,44^

### 4.3. Electrochemical studies

**Fig. 8:**
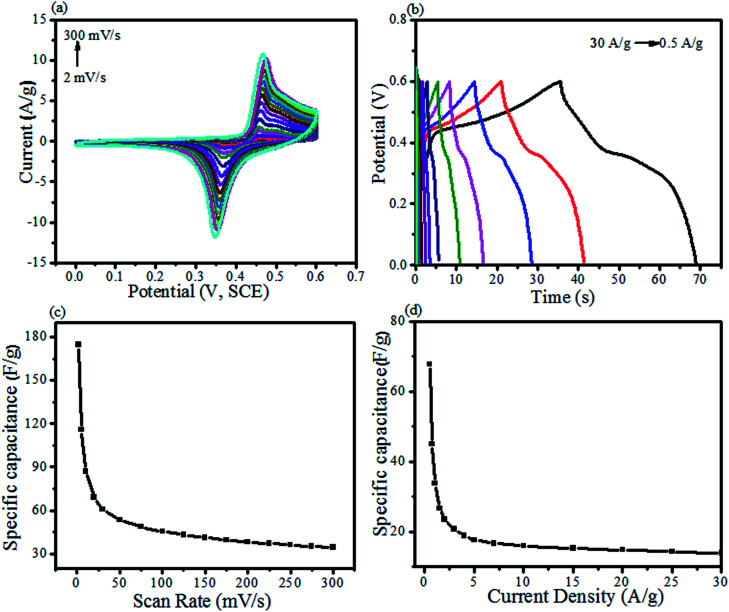
Supercapacitive properties of the ZnO@PdO/Pd electrode by (a) CV at applied scan rates (ASR), (b) GCD measurements at applied CD, (c) SC of ZnO@PdO/Pd electrode at ASR, and (d) SC at applied CD.

**Fig. 9:**
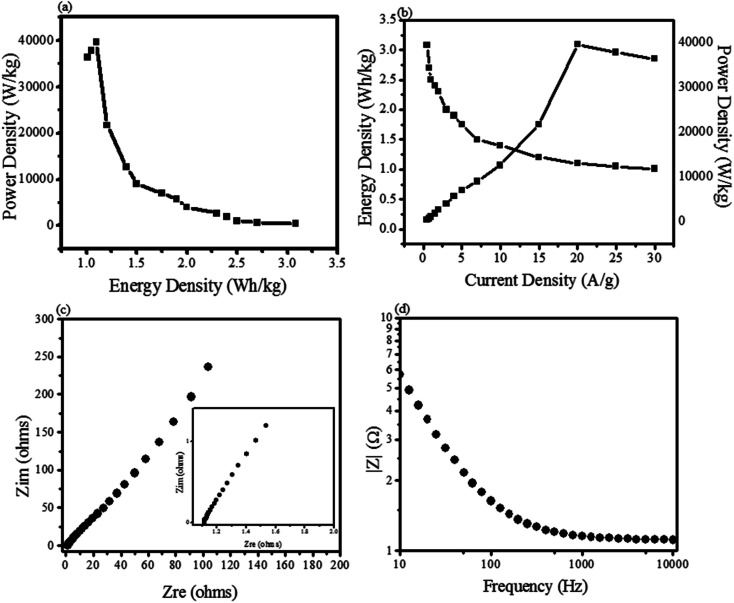
(a) Ragone plot by ZnO@PdO/Pd; (b) measured ED and PD on different CD; (c) Nyquist plot by ZnO@PdO/Pd at low frequency part (inset: Nyquist plot at higher frequency part); (d) change in impedance along with frequency.

The reported literature presents PdO as an excellent electrochemical test material having precise redox behavior.^72–74^ Moreover, it can ominously lower the BGE of ZnO, resulting in a boosted electrochemical behavior (Fig. 8 and 9). In our previous study, we have synthesized ZnO NPs employing the phytochemical extract for supercapacitive investigation.^5^ In the current study, the effects of Pd/PdO were investigated for the reported electrochemical behavior of ZnO NPs.^5^

Fig. 8a and b reveal CV as well as GCD profiles given by ZnO@PdO/Pd, pointing Faradaic redox peaks by pseudocapacitors. With an upsurge in SR from 2–300 mV s^−1^, anodic (AP) and cathodic peaks (CP) altered their intensity, as well as the positions. AP showed an increase in CD (peak), while moving to high potentials. However, CP showed a decline in CD showing a shift to low potentials. These results were in agreement with those obtained by Khan *et al.*,^32^ Duraisamy *et al.*^34^ and Pramanik *et al.*^35^ Reasons behind these findings were kinetic irreversibility as well as electric polarization given by ions of electrolyte at the surface of the electrode with higher SR.^33,36–40^ This changed the CV curves shape to a rectangular form with more SR. SC values shown by the ZnO@PdO/Pd electrode are noted at different SR (Fig. 8c). SC of 178 F g^−1^ were witnessed at about 2 mV s^−1^ by CV data (Fig. 8c), indicating efficient stability of ZnO@PdO/Pd that maintained high at 2 mV s^−1^. However, for ZnO NPs, 86 F g^−1^ was recorded at 2 mV s^−1^.^5^ As discussed above, Pd has a fast redox behavior,^74^ and it is well reported to have fast redox reactions, contributing to the enhancement of the specific capacitance.^40–50^Table 1 shows a contrast of the capacitance shown by the ZnO@PdO/Pd electrode with those obtained in earlier reports.

**Table 1:** Contrast between the as-synthesized ZnO@PdO/Pd electrode and earlier reports

Electrode	Electrolyte	Specific capacitance (F g^−1^)	Scan rate (mV s^−1^)	References
ZnCo_2_O_4_ nanorods	PVA	10	10	45
		5	100	
ZnCo_2_O_4_ nanowires	PVA	0.4 mF	100	46
		0.85 mF	30	
ZnO–Co_3_O_4_	3M KOH	165	2	1
Co_3_O_4_ powder (<50 nm)	2M KOH	118	5	10
Co-doped TiO_2_NT/RGO	Na_2_SO_4_	34.8	5	49
ZnO NPs	3M KOH	86	2	5
ZnO@PdO/Pd	3M KOH	178	2	Present work
		118	5	
		57	100	

Fig. 8 shows the charge storage capacity of M.Os composite investigated by GCD measurements. Non-linear curves are observed in GCD, which agreed with the observation from CV data, confirming the pseudocapacitance behavior of ZnO@PdO/Pd. Extreme SC attained by GCD data was about 69 F g^−1^ at 0.5 A g^−1^ (Table 2).

**Table 2:** Comparison of the GCD-based capacitance of ZnO@PdO/Pd with literature

Electrodes	Electrolytes	SC value (F g^−1^)	CD A g^−1^	References
ZnO–Co_3_O_4_	3M KOH	84.3	0.5	1
Ti_3_C_2_–ZnO	KCL	70	0.5	51
ZnO	3M KOH	39	0.5	5
ZnO@NiO	Aqueous KOH	34	5	3
Mo–ZnO	KOH	46.2	10	50
ZnO@PdO/Pd	3M KOH	69	0.5	Current work

The ability of energy storage shown by ZnO@PdO/Pd was estimated *via* specific ED (W h kg^−1^) along with PD (W kg^−1^) (Fig. 9a and b). Fig. 9b shows that ZnO@PdO/Pd exhibited the highest ED, *i.e.*, 3.3 W h kg^−1^, which also is better than previous findings on electrodes of ZnO,^39^ ZnO,^5^ MoO_2_, Mo O_3_ (ref. 66 and 69) and Co_3_O_4_.^56^ The ZnO NPs revealed the highest ED of 1.9 W Kg^−1^,^5^ which is much lower than ZnO@PdO/Pd. This is again credited to the fast Faradaic reactions of PdO/Pd.^55–67^ ED of PF-binded ZnO@PdO/Pd was also improved than ZnO-activated carbon^39^ ZnO NPs.^40^ Excellent PD of ZnO@PdO/Pd was 3718 W kg^−1^ (3.7 KW kg^−1^) (Fig. 9b), which is much better than ZnO NPs,^5^ ZnO,^39^ ZnO–Co_3_O_4_ (ref. 1) and ZnO@NiO.^3^Fig. 9a shows a Ragone plot revealing a negative relation among ED and PD, as verified in previous findings.^29–32^ Accordingly, both ED and PD demonstrated ZnO@PdO/Pd as an efficient electrode for energy storage devices.

EIS measurements were also carried out to evaluate the internal resistance (*R*_i_) as well as the charge transfer resistance (*R*_ct_), as shown in Fig. 9c and d. The inset shown in Fig. 9c in Nyquist displayed a semicircle at lower frequencies, pointing to *R*_ct_. Intercept (in real part) shown at higher frequencies was because of *R*_i_. Fig. 9a shows a line at 45° that is known as the Warburg element (*Z*_w_).^1,29,30^ Lower *R*_i_ of 0.4 Ω shown by our prepared ZnO@PdO/Pd confirmed the high speed movement of ions as well as electrons, and this low value is because of more C and O consisting OFGs at the electrode surface, thus creating more sites for the transportation of electrons and ions giving a rise to improved conductivity.^50–55,67–70^ The semi-circle arc shown in Fig. 9c verified the best supercapacitor behavior also authenticated by a vertical line shown by *Z*_w_ as well as a minor semicircle present along with it. Fig. 9d represents a very low impedance along with a change in frequency also verified the best conductivity given by ZnO@PdO/Pd.

These electrochemical examinations including the low *R*_i_ and *R*_ct_ showed a superb redox behavior (CV as well as GCD), verifying that the fabricated ZnO@PdO/Pd nanostructure can be fruitfully employed as a supercapacitor.

## 5. Conclusion

We have successfully synthesized the facile ZnO@PdO/Pd nanomaterial demonstrating its pretrial utility for energy storage. The ZnO@PdO/Pd nanomaterial revealed a spherical shape and less agglomeration. XPS analysis and GCMS unveiled ZnO@PdO/Pd surface functionalization by phyto compounds (C_8_H_11_N, C_7_H_8_O, and C_8_H_19_N). Consequently, ZnO@PdO/Pd displayed more active sites, thus facilitating the effective diffusion of electrons as well as ions. An enhanced specific capacitance and energy density was obtained for the fabricated electrode due to the combination of ZnO and PdO. The significant redox behavior was noticed in the fabricated electrode, as depicted *via* CV along with GCD, revealing that it had a considerable potential for energy storage devices. Thus, current investigation presented the excellent potential of ZnO@PdO/Pd for pseudocapacitance.

## Conflicts of interest

There are no conflicts of interest among the authors.

## Supplementary Material
